# 急性B淋巴细胞白血病CD19 CAR-T细胞治疗后阳性复发的机制和挽救性治疗策略

**DOI:** 10.3760/cma.j.cn121090-20240701-00242

**Published:** 2024-10

**Authors:** 聪 卢, 佳 徐, 恒 梅

**Affiliations:** 华中科技大学同济医学院附属协和医院血液科，湖北省肿瘤疾病细胞治疗临床医学研究中心，武汉 430022 Institute of Hematology, Union Hospital, Tongji Medical College, Huazhong University of Science and Technology, Hubei Clinical Medical Center of Cell Therapy for Neoplastic Disease, Wuhan 430022, China

## Abstract

尽管嵌合抗原受体T（CAR-T）细胞疗法改变了复发/难治急性B淋巴细胞白血病（R/R B-ALL）的治疗格局，仍然有50%患者在治疗后1年内复发，且60％左右为阳性复发。导致CAR-T细胞治疗复发的机制主要与肿瘤异质性、CAR-T细胞自身功能障碍和体内扩增及持续性不佳以及抑制性免疫微环境相关。这篇综述旨在探讨改善CAR-T治疗后复发或进展患者结局的挽救性策略，包括同一靶点二次CAR-T细胞治疗或者更换靶点回输，联合免疫检查点抑制剂，新型靶向药物包括新型单克隆抗体、抗体药物偶联物以及双特异性抗体等应用，使用小分子化合物逆转CAR-T耐药等，阐述了可能的相关机制并列举了相关的临床资料。经过对患者CAR-T治疗失败后的复杂情况探讨，我们认为，这些药物在用于复发时的挽救性治疗、CAR-T治疗后维持治疗或在CAR-T细胞回输前或者回输时联合治疗时，在一定程度上可减少阳性复发或克服耐药，但改善患者的长期生存仍然面临巨大挑战。

## Abstract (en)

Approximately 50% of patients suffering from relapsed/refractory B-acute lymphoblastic leukemia（R/R B-ALL）, experience relapse within one year, with around 60% of these relapses being antigen-positive, despite the transformative impact of chimeric antigen receptor（CAR）T cell therapy. The mechanisms underlying relapse are primarily associated with tumor heterogeneity, CAR-T cell dysfunction, subopimal in vivo expansion and persistence, and an inhibitory immune microenvironment. This review aims to investigate salvage strategies designed to enhance outcomes for patients undergoing relapse or disease progression following the CAR-T cell therapy. These strategies include a second CAR-T cell infusion that targets either the same antigen or an alternative target, the administration of immune checkpoint inhibitors, and the utilization of novel targeted therapies including monoclonal antibodies, antibody-conjugated drugs and small molecule compounds aimed at mitigating CD19-positive relapse or overcoming CAR-T cell resistance. Nevertheless, achieving improved long-term survival for these patients continues be challenging.

抗CD19嵌合抗原受体T（chimeric antigen receptor T, CAR-T）细胞治疗显著提升复发/难治急性B淋巴细胞白血病（R/R B-Acute lymphoblastic leukemia, B-ALL）的疗效，但治疗后短期复发严重影响患者的长期生存。目前关于CAR-T治疗后复发主要可分为三种类型：CD19阳性（CD19pos）复发、CD19阴性（CD19neg）复发以及谱系转换[1]。研究表明，B-ALL患者接受CD19 CAR-T治疗后复发中CD19pos比例高达59％～92％[2]–[5]。尽管目前CAR-T桥接异基因造血干细胞移植是减少复发的有力手段，但临床中存在CAR-T前移植史、移植相关严重并发症及多线治疗导致的严重免疫缺陷等问题，使得部分患者无法从桥接移植中获益。因此阐明阳性复发机制和寻找有效的复发后治疗策略是目前亟待解决的问题。

在本综述中，我们总结了目前CAR-T细胞治疗后阳性复发的可能机制和复发后可供选用的治疗方案。影响CD19 CAR-T患者复发风险的因素包括肿瘤负荷、缓解程度、CAR结构和CAR-T细胞在体内的扩增和持续性等。CAR-T细胞二次输注、使用包括双特异性抗体在内的小分子药物和其他挽救性策略仍然可以给这部分患者带来希望。

一、阳性复发的机制探讨

（一）患者回输前疾病情况

CD19pos和CD19neg复发的决定因素尚不明确。在一项研究中，对163例接受CD19 CAR-T治疗后复发的患者进行了回输前相关因素的分析[6]，有83例（50.9％）为CD19 pos。多变量Cox回归模型中，≥2次既往缓解是唯一与CD19 pos复发风险增加的独立相关变量。然而4-1BB CAR结构、既往Blinatumomab治疗无应答和CAR-T治疗前高肿瘤负荷（>5％原始细胞）与CD19neg复发风险增加相关。Dourthe等[7]设计了前瞻性队列研究，共纳入了51例接受Tisagenlecleucel输注的儿童和年轻成人R/R B-ALL患者。数据显示，清淋前低肿瘤负荷（*P*＝0.03）和过早恢复的B细胞缺乏（B-Cell aplasia, BCA）（*P*＝0.004）与CD19pos复发风险增加相关。所有患者在第28天均同时发生BCA，至BCA恢复的中位时间为7.7个月。单变量分析BCA恢复唯一预测因素是清淋前低肿瘤负荷。

（二）CAR-T细胞功能及体内扩增存续能力

1. 患者自身来源的T细胞质量不佳：T细胞质量是CAR-T细胞持久性的决定因素之一，既往多线化疗及放疗可无差别地对免疫细胞进行攻击进而影响其功能。化疗药物和放疗可导致T细胞凋亡、抑制T细胞亚群增殖和分化，从而降低T细胞质量，如含有氯法拉滨或阿霉素的化疗可造成淋巴细胞减少[8]。研究表明，富集早期谱系细胞的T细胞群体扩增更好，而在儿童ALL和非霍奇金淋巴瘤（NHL）患者化疗期间对CAR-T细胞扩增和记忆表型的研究发现，环磷酰胺和阿糖胞苷会耗尽早期谱系细胞，从而导致CAR-T细胞治疗不佳[8]。

2. CAR-T细胞亚群：多项研究表明CAR-T细胞亚群的比例对CAR-T长期存续及患者预后有重要影响。复发患者中的CAR-T细胞缺乏中央记忆（CD45RO^+^、CD62L^+^、CD95^+^）或干细胞样记忆T细胞（CD45RA^+^、CD62L^+^、CD95^+^）[9]。Bai等[10]通过单细胞测序的方法分析了费城儿童医院R/R B-ALL的1/2期CD19 CAR-T细胞临床试验中的12例儿童和年轻成人患者，发现CD19pos复发与Th2功能缺陷、CAR-T细胞维持早期记忆状态的能力不足有关，长期完全缓解（CR）患者中编码Th2功能的所有主要基因，包括IL-13、IL-5、IL-4和上游调节因子GATA3均上调。

最近一项对大B细胞淋巴瘤（LBCL）患者细胞输注产品研究发现，Tisa-cel的疗效与产品中的CD8^+^中央记忆样细胞的显著扩增有关，Tisa-cel以及Axi-cel两种CAR-T产品中的CAR-Treg细胞可以抑制CAR-T细胞的活性，从而促进复发[11]。研究者对11例CD19 CAR-T治疗后的B-ALL患儿进行了详细的CAR-T细胞表型分析发现，一些长期存在的CAR-T细胞具有双阴性αβT细胞群，不表达CD8-α或CD4共受体。这些细胞在晚期CAR-T细胞中占主导地位，并表现出活化、增殖和效应状态，与早期CAR-T细胞群相比具有更高的活化标志物表达，并表达与细胞记忆相关的基因[12]。在另一项最新研究58例接受Axi-cel治疗R/R弥漫大B细胞淋巴瘤（DLBCL）的患者，CR与部分缓解/未缓解（PR/NR）患者相比，血液中CAR-T细胞持久性更长，而CAR-Tγδ^+^细胞数（*OR*＝8.8）和CAR-Th1TM细胞数（*OR*＝6.2）为CR的独立预测因子[13]。

3. CAR结构中共刺激分子的影响：共刺激分子包括CD28、ICOS、CD27、4-1BB、OX40和CD40L等。包含4-1BB共刺激域的CAR-T细胞中位持续时间更长，而包含CD28共刺激域的CAR-T细胞输注后扩增峰值出现时间更早。CAR-T细胞持久性及快速扩增两者中哪一个对于疗效具有更关键的作用，还有待进一步研究[14]。研究报道含有4-1BB的CAR-T细胞通过效应记忆T细胞（Tem）表型标志物增加，以及特异的下游分子活化途径延迟CAR-T细胞耗竭和凋亡[15]。而CD19-28ζ CAR-T细胞表现出转录因子NF-κB的活化增加，而NF-κB负责细胞因子基因转录和分泌的诱导[16]。因此，这些细胞具有高细胞毒性和扩增的特点，但也由于CAR单链抗体可变区（scFv）聚集导致的过度的胞内信号域CD3ζ磷酸化而出现快速耗竭[17]，同时由于其高细胞毒性和扩增能力，导致了严重细胞因子释放综合征（CRS），这部分患者可能需要类固醇治疗，而后者又限制了CAR-T细胞的持久性。而含有4-1BB共刺激域CAR-T细胞表现出更好的持久性，耗竭和凋亡相较含CD28共刺激域CAR-T细胞有所改善[18]。

4. 免疫原性：人类免疫系统对于鼠源CAR的scFv具有天然的免疫排斥作用，这也是鼠源CAR-T治疗后复发的机制之一。临床研究中使用的抗CD19单链抗体可变区大多来源于小鼠，由于其高抗原性，这可能导致患者的CAR-T细胞衰竭，从而导致阳性复发[19]。人源化scFv可减少CAR-T细胞的抗原性来增强其体内持久性以提高其功效[20]。

（三）免疫抑制微环境

CAR-T治疗期间由于程序性细胞死亡蛋白1（PD-1）等抑制性分子表达增加会使得CAR-T细胞寿命缩短。这些抑制性分子受体正是在效应T细胞发挥作用产生细胞毒性反应中诱导的[21]。处于疲惫状态的T细胞高表达抑制性受体，还包括T细胞免疫球蛋白和黏蛋白结构域包含蛋白3（TIM-3）、淋巴细胞活化基因3（LAG-3）、CD160、B和T淋巴细胞衰减因子（BTLA）、细胞毒性T淋巴细胞相关抗原4（CTLA-4）和T细胞免疫受体Ig和ITIM结构域（TIGIT）等。免疫应答的协同抑制因子，与表达在T细胞表面的上述配体结合，可调节T细胞的活性，激活效应T细胞的凋亡，抑制调节性T细胞（Treg）的凋亡，从而促进自身耐受[22]，使得T细胞增殖、细胞因子分泌减少，持久性变差。

二、挽救性治疗策略

目前的挽救性策略主要包括二次CAR-T治疗、免疫检查点抑制剂（ICI）、新型靶向治疗药物等以及小分子化疗药物联合治疗等。

（一）二次CAR-T治疗

在一项88例B-ALL患者接受Tisa-cel（鼠源CD19 CAR-T）治疗的研究中，84.6％的患者体内产生了针对CAR的抗体。对产生抗CAR抗体的患者进行二次CAR-T细胞回输时，CAR-T细胞的扩增通常较微弱，且持续时间不长。即使添加了IL-2来尝试改善CAR-T细胞扩增问题，疗效仍不尽如人意。鼠源CD19 CAR-T细胞疗法的二次回输完全缓解率仅为25％[23]。因此，对于临床免疫源性处置的建议是更换为没有交叉免疫源性的CAR载体，比如鼠源换人源化，人源化换全人源等。

1. CD19 CAR-T细胞二次输注：Gauthier等[24]为探索输注CD19 CAR-T细胞（鼠源FMC63）治疗失败的患者再次输注的疗效以及影响因素，共纳入44例患者进行分析，大多数患者二次CAR-T剂量高于初次CAR-T，且回输前进行了再次淋巴细胞清除。研究结果显示总体有效率39％，21％的B-ALL患者达CR，中位随访28个月，中位反应时间为4个月。Holland等[25]回顾性分析了儿童和年轻成人B-ALL患者再次回输相同的鼠源CD19 CAR-T产品的应答性和毒性反应，同时评估了两次CAR-T回输之间的CAR-T细胞扩增和白血病抗原表达情况。18例患者在初次CAR-T治疗后因持续肿瘤抗原阳性（7例）或复发（11例）进行了二次回输。18例患者中，7例（38.9％）获得CR/PR，其中5例达到微小残留病（MRD）阴性CR，1例MRD阳性CR，1例PR，应答者包括4例初次CAR-T治疗耐药患者。研究显示二次CAR-T回输后CRS并不剧烈，且外周血CAR-T扩增峰值二次回输低于初次回输（*P*＝0.03）。6例（33.3％）患者在接受二次CAR-T治疗后出现肿瘤抗原的丢失或下调，导致患者未能获得CR。此研究也表明虽然部分患者对初次CAR-T应答不佳或发生复发，但他们仍可从二次CAR-T治疗中获益。强化清淋方案可能是增强二次CAR-T治疗应答的策略之一。

2. 更换靶点治疗：虽然CD19 CAR-T在B-ALL中的缓解率较高，但为克服复发耐药问题，仍然值得探索新的靶点。CD22在成人和儿童B-ALL中的表达率约为90％[26]。Fry等[27]报告了一项Ⅰ期临床试验的结果，该试验纳入21例儿童和成人，其中包括17例既往接受过CD19 CAR-T治疗，患者均接受CD22 CAR-T新型细胞治疗，接受≥1×10^6^/kg CD22 CAR-T细胞治疗的15例患者中，73％（11/15）达到CR，其中包括达到缓解的6例CD19pos和5例CD19dim/neg的B-ALL患者。Li等[28]收集5例接受靶向CD19 CAR-T治疗后复发的患者，分别培养CD19和CD22 CAR-T细胞，以大致1∶1混合。再次回输CAR-T后，全部5例患者均达到MRD阴性CR。中位随访时间26.3个月，6、12个月总生存（OS）率均为100％。而在上述临床研究中，更换靶点后的缓解率达72.9％，显著高于同一靶点[25]。此外，Pan等[29]采用序贯输注CD19和CD22 CAR-T的策略来阻止肿瘤免疫逃逸，并延长CAR-T细胞的持久性。

（二）ICI

长期抗原暴露会导致CAR-T细胞耗竭状态，并伴随抑制性的肿瘤免疫微环境（TME）[30]。研究发现，在对CAR-T细胞无反应的患者中，肿瘤细胞和TME中其他细胞的抑制性检查点蛋白表达（如PD-L1、LAG3）的基线水平更高。因此，通过使用免疫检查点抑制剂，可以阻止免疫检查点蛋白与其配体结合发挥抑制作用，提高CAR-T细胞的增殖能力，增强CAR-T细胞的细胞毒性，延长CAR-T细胞在体内的存活时间从而再次激活CAR-T细胞，提高治疗效果[31]。

Li等[32]的研究纳入费城儿童医院接受过靶向CD19 CAR-T细胞治疗（鼠源CTL019或人源化CTL119）并在治疗后早期表现出无应答或者部分应答的14例B-ALL患者，在CAR-T细胞输入后14 d内和CRS症状消退后开始给予PD-1抑制剂帕博利珠单抗治疗，最多每3周重复给药1次。研究发现，其中6例出现早期BCA患者中有5例恢复。4例患者为CAR-T治疗后无反应或复发后伴随大体积髓外病灶，在接受PD-1抑制剂治疗后2例分别达到PR和CR。部分患者在开始使用帕博利珠单抗后数日内检测到显著的CAR-T细胞增殖，并且与影像学疾病缓解呈时间相关性。T细胞耗竭或活化诱导的CAR-T细胞死亡导致CAR-T细胞持久性减弱，而ICI应用于复发性B-ALL患者，可增强CAR-T细胞杀肿瘤效应和持久性。Maude等[33]为研究PD-1抑制剂对CAR-T细胞扩增、功能和持久性的改善作用，对4例接受鼠源CTL019或人源化抗CD19 CAR-T细胞治疗且在14 d到2个月内无缓解或者出现进展的R/R B-ALL患儿，进行1～3次帕博利珠单抗治疗。结果显示帕博利珠单抗延长了4例患儿循环CAR-T细胞的检出时间，客观缓解率为50％（2/4）。其中1例于CAR-T输注后28 d出现髓外复发的患者在3个月PET-CT复查提示肿瘤负荷显著减小，所有患者均耐受性良好。由此证明帕博利珠单抗安全性较好，同时可逆转CAR-T细胞耐药性，重启CAR-T扩增的存续。

（三）新型靶向治疗药物

1. 单克隆抗体：单克隆抗体可提升CAR-T细胞疗效，但其在CAR-T治疗复发后的使用尚在探索中。在一项针对R/R B-ALL的研究中，在CAR-T细胞治疗中加入利妥昔单抗显著改善了长期结局，28 d内80％患者达到了MRD阴性CR，而仅接受CAR-T细胞治疗的对照组CR率为60％，联合治疗组的2年OS率也更高（90％对26.7％）[34]。坦昔妥单抗（Tafa）和朗妥昔单抗（Lonca）分别为靶向CD19的单克隆抗体和ADC药物，一项为评估这些靶向疗法在治疗CD19 CAR-T治疗后复发LBCL患者疗效的研究共纳入53例患者，36例评估了疾病复发时CD19表达，CD19pos占61％（22例）；40例（75％）患者接受坦昔妥单抗±来那度胺治疗，13例（25％）接受朗妥昔单抗治疗，两组的总缓解率和CR率分别为23％、36％和7％、18％[35]，提示这些靶向疗法在CAR-T治疗后疾病复发患者中的疗效仍然有限。

2. 抗体偶联药物（ADC）：

（1）CD22 ADC：CD22抗体（inotuzumab ozogamicin）是由靶向CD22人源化单抗和细胞毒性药物卡奇霉素组成的ADC。药物与肿瘤细胞表面CD22抗原结合后迅速内化，卡奇霉素在溶酶体中被释放出来，导致细胞DNA双链断裂和细胞凋亡，从而起到杀伤肿瘤细胞的作用[36]。安丽红等[37]的回顾性研究共纳入了2020年3月至2022年9月接受两剂CD22 ADC治疗并评估了疗效的21例R/R B-ALL患者，其中18例患者既往接受过CD19 CAR-T或者CD19/CD22双靶CAR-T治疗，21例患者中有14例（66.7％）获得CR，显示良好的缓解率。

（2）CD19 ADC：一项剂量递增、扩展临床试验评估了朗妥昔单抗治疗成人R/R B-ALL的安全性和耐受性[38]，共纳入35例R/R B-ALL患者，其中有2例接受过CAR-T治疗，所有患者均接受至少1次每3周1次15～150 mg/kg的朗妥昔单抗，由于研究提前终止，尚未对疗效规范评估，只在低中高三个剂量组中各评估了1例患者，均达到CR，且3例患者中有两例治疗前接受过贝林妥欧单抗的治疗，疗效分别为PR和NR，但接受一定周期朗妥昔单抗治疗后可达CR。遗憾的是未对CAR-T治疗复发的患者进行疗效评估。

3. 双特异性抗体：双特异性T细胞衔接器（BiTE），通过靶向肿瘤细胞和T细胞上的抗原，介导免疫突触形成，诱导T细胞活化和增殖，已被用于探索CAR-T治疗B细胞恶性肿瘤复发的后续治疗。CD19 BiTE在CD19pos复发中可能具有潜在疗效。贝林妥欧单抗是第一个用于临床的双特异性抗体药，批准用于治疗R/R的B-ALL。Qi等[39]探讨了贝林妥欧单抗作为挽救治疗在CD19 CAR-T细胞治疗失败R/R B-ALL患者中的疗效及安全性，共收集了5例患者，CD19均为部分或高表达（32％～99％），在使用1～2周期静脉输注贝林妥欧单抗后评估，有4例患者达到了CR。

三、小分子化合物参与的维持治疗或联合治疗策略

小分子化合物可通过各种机制逆转耗竭的CAR-T细胞，增强其抗肿瘤活性，大多被直接用于CAR-T回输后的维持治疗[40]。CD19 CAR-T二次回输作为临床可及性较好的挽救性治疗手段，也可同时联合小分子化合物。

1. 来那度胺：来那度胺（Lenalidomide, LEN）属于免疫调节剂，其主要从下列机制调控CAR-T细胞活性和功能，包括促进T细胞与肿瘤细胞间的免疫突触形成、降低免疫检查点分子对T细胞的免疫抑制作用、促进共刺激阈的酪氨酸磷酸化激活T细胞、促进T细胞分泌IL-21增加记忆T细胞亚群等。临床前研究发现，来那度胺可促进CAR-T细胞增殖，减少CAR-T细胞耗竭，同时还可通过刺激细胞毒性CD8^+^ T细胞扩增来增强其抗肿瘤活性[41]。王一等[42]收集了本院38例经CAR-T治疗后达到CR的B-ALL患者，18例（47.4％）于治疗3～6个月后采用了以来那度胺为基础的维持治疗方案，分析显示CAR-T治疗后接受维持治疗是OS和无白血病生存（LFS）的保护因素，可以使患者获得更好的长期生存。苏燕等[43]报道了1例老年难治B-ALL患者，CD19 CAR-T细胞回输体内后达3个月仍未被检出，后经那度胺联合间断IL-2治疗，4个月后CAR拷贝数再次上升，该例患者至随访45个月时仍然维持持续缓解状态。

2. 达沙替尼：酪氨酸蛋白激酶抑制剂达沙替尼能有效抑制T细胞活化，最近的研究表明，达沙替尼能够通过对抗TCR信号通路，干扰淋巴细胞特异性蛋白酪氨酸激酶的合成抑制CD3ζ和T细胞受体相关蛋白激酶70kDa（ZAP70）ζ链的磷酸化，限制活化T细胞核因子（NFAT）的诱导功能，从而减少CAR信号下行。在实验中，接受达沙替尼处理的CAR-T细胞表现出更好的增殖、较低水平的抑制性受体表达和功能恢复，更好地控制肿瘤生长。在临床CAR-T细胞生产环境中使用达沙替尼可以缓解有害CAR信号传导，重新激活耗竭的CAR-T细胞[44]。米瑞华等[45]报道1例确诊5年的R/R Ph^+^ B-ALL的53岁女性患者，CD19 CAR-T治疗达CR后半年后再次出现MRD和分子学复发后使用贝林妥欧联合达沙替尼治疗，治疗第15天复查骨髓情况提示再次转为阴性，之后院外口服达沙替尼，随访6个月以上仍持续缓解中。

3. 地西他滨：地西他滨作为甲基转移酶抑制剂，可修改耗竭相关DNA甲基化程序来改善CAR-T细胞功能。研究发现，向B-ALL患者体内输注4-1BB CD19 CAR-T细胞，在4周后获得了耗竭相关的DNA甲基化特征，而敲除CAR-T细胞的DNMT3A基因，能够防止T细胞耗竭，并增强抗肿瘤活性[46]。Wang等[47]的研究显示，CAR-T制备期加入地西他滨可有效改善CAR-T细胞耗竭、增强CAR-T细胞增殖能力及抗肿瘤活性。转录组测序结果显示，与单独应用CAR-T细胞相比，在联合治疗方案中，记忆和增殖相关基因的表达上调，T细胞抑制因子、死亡和活性/耗竭相关基因的表达下调增强。Ma等[48]开展了一项针对CD19/CD22 CAR-T细胞治疗后患者开展维持治疗的研究，纳入48例接受过CD19/CD22 CAR-T细胞治疗达到CR的R/R B-ALL患者，其中16例采取地西他滨作为维持治疗，32例未进行维持治疗（对照组）。研究结果显示，地西他滨组和对照组的3年OS率分别为90.9％对66.1％；LFS率分别为81.3％对61.3％，表明CAR-T细胞治疗后接受地西他滨可改善R/R B-ALL患者预后。

4. 布鲁顿酪氨酸激酶抑制剂（BTKi）：BTKi可以明显改善T细胞的功能缺陷并且增强自身T细胞在体外扩增的能力，并且使与CAR-T细胞增殖能力呈负相关的T细胞上抑制受体PD-1和CD160以及免疫抑制分子CD200的表达降低[49]。Im等[50]进行的一项研究表明CD19 CAR-T细胞与表达CD19 B-ALL细胞之间相互作用导致所有白血病细胞CD19聚集和内化，从而导致CD19表面密度降低；且单细胞测序证明了CD19low细胞通过生发中心反应和生发中心激活的转录程序持续降低CD19表达；而使用BTKi伊布替尼预处理白血病细胞，可以抑制转录程序的启动，提高CD19 CAR-T细胞对肿瘤细胞的有效识别和杀伤。

5. 其他小分子化合物：选择性核输出抑制剂（SINE）、组蛋白去乙酰化酶抑制剂（HDACi）、磷脂酰肌醇-3-激酶（PI3K）抑制剂以及Bcl-2抑制剂等小分子化合物均在体外试验中被证实可增强CAR-T抗肿瘤效应。Wang等[51]研究发现CD19阳性肿瘤细胞可被Eltanexor和塞利尼索杀伤，且其对SINE的敏感性与ALL细胞系中XPO1的表达水平相关，依次使用SINE和CAR-T细胞可能会从整体上提高CAR-T细胞的抗肿瘤功效。Torres-Collado等[52]发现，当人类NHL细胞系逐渐对CD19 CAR-T细胞治疗产生耐药时，HDACi逆转了对CD19 CAR-T细胞治疗的耐药性。Zheng等[53]研究发现，在CAR-T细胞体外扩增过程中，PI3K抑制剂可促进记忆细胞形成，增强CAR-T抗肿瘤效应。Yang等[54]研究证实，当肿瘤细胞被Venetoclax预致敏时，CD19 CAR-T细胞的杀伤效率显著提高。这些药物在B-ALL中与CAR-T联合治疗的作用仍待进一步研究和探索。

四、总结

总之，靶向CD19的CAR-T免疫疗法对R/R B-ALL患者具有很高的缓解率，但1年内的复发率仍有50％左右。这些患者的预后通常较差，而目前对于CAR-T治疗失败的患者也尚无标准治疗方案，因此迫切需要挽救性药物单药或联合CAR-T来治疗这些复发的患者。CAR-T细胞治疗失败后，确定适当的治疗方案需要考虑患者的一般情况、治疗期的长短、药物的可及性等多个因素。而对于这些药物在复发时的挽救性治疗，或在CAR-T治疗后维持期治疗，或者与CAR-T回输时进行联合治疗，对CAR-T细胞的扩增能力和持久性的潜在影响，对阳性复发率的降低和对患者长期生存的改善程度，仍然是值得进一步探索的话题。
